# Lipidomics Indicates the Hepatotoxicity Effects of EtOAc Extract of *Rhizoma Paridis*

**DOI:** 10.3389/fphar.2022.799512

**Published:** 2022-02-08

**Authors:** Chaofeng Li, Mingshuang Wang, Tingting Fu, Zhiqi Li, Yang Chen, Tao He, Dan Feng, Zhaoyi Wang, Qiqi Fan, Meilin Chen, Honggui Zhang, Ruichao Lin, Chongjun Zhao

**Affiliations:** ^1^ Beijing Key Lab for Quality Evaluation of Chinese Materia Medica, School of Chinese Materia Medica, Beijing University of Chinese Medicine, Beijing, China; ^2^ School of Chinese Materia Medica, Beijing University of Chinese Medicine, Beijing, China

**Keywords:** Lipidomics, *Rhizoma Paridis*, hepatic Fibrosis, DILI, traditional Chinese Medicine

## Abstract

*Rhizoma Paridis* is a traditional Chinese medicine commonly used in the clinical treatment of gynecological diseases. Previous studies have shown that aqueous extracts of *Rhizoma Paridis* exhibit some hepatotoxicity to hepatocytes. Here, using lipidomics analysis, we investigated the potential hepatotoxicity of *Rhizoma Paridis* and its possible mechanism. The hepatic damaging of different solvent extracts of *Rhizoma Paridis* on zebrafish larvae were determined by a combination of mortality dose, biochemical, morphological, and functional tests. We found that ethyl acetate extracts (AcOEtE) were the most toxic fraction. Notably, lipidomic responsible for the pharmacological effects of AcOEtE were investigated by Q-Exactive HF-X mass spectrometer (Thermo Scientific high-resolution) coupled in tandem with a UHPLC system. Approximately 1958 unique spectral features were detected, of which 325 were identified as unique lipid species. Among these lipid species, phosphatidylethanolamine cardiolipin Ceramide (Cer), lysophosphatidylinositol sphingosine (Sph), etc., were significantly upregulated in the treated group. Pathway analysis indicates that *Rhizoma Paridis* may cause liver damage *via* interfering with the glycerophospholipid metabolism. Collectively, this study has revealed previously uncharacterized lipid metabolic disorder involving lipid synthesis, metabolism, and transport that functionally determines hepatic fibrosis procession.

## Introduction

Drug-induced liver injury (DILI), which can be caused by conventional clinical drugs, herbal medications, and dietary supplements (Kuna et al., 2018; Tillmann et al., 2019), is also the most common cause of acute liver failure in the United States and Europe (Fontana et al., 2014). DILI can be affected by many factors, such as gender, age, ethnicity, pregnancy, alcohol consumption, and their interaction (Kumar et al., 2021). Therefore, early identification of DILI is essential primarily to improve drug safety and reduce the cost of drug development.

In a state of persistent liver injury (metabolic, cholestatic, or toxic), hepatocellular damage triggers a series of events that culminate in the activation of quiescent hepatic stellate cells (HSC) to a myofibroblastic (activated) HSC state (Mederacke et al., 2013). Multiple pathways direct HSC activation and ultimately leads to increased secretion of extracellular matrix proteins such as collagens that eventually accumulate in the liver parenchyma and lead to liver fibrosis (Friedman, 2008). Hepatic fibrosis is a repair response to diffuse over deposition and abnormal distribution of extracellular matrices such as glycoproteins, collagen, and proteoglycans after various injuries to the liver and is a critical step in the progression of various chronic liver diseases to cirrhosis. Although there have been many advances in basic and clinical research on liver fibrosis in recent years, there is still a lack of ideal drugs to treat liver fibrosis.

*Rhizoma Paridis*, which has significant hemostatic and anti-tumor effects, is the main rhizome of *Paris Polyphylla* var *yunnanensis* (Franch.) Hand. -Mazz, or *Paris Polyphylla* Smith var. *chinensis* (Franch.) Hara (Qin et al., 2018). Relevant studies have shown that *Rhizoma Paridis* is hepatotoxic. Polyphylin I, II, VI, and VII were cytotoxic to both hepatocytes-HL-7702 and HepaRG cells. Furthermore, Polyphylin I, which can induce HepG2 cells’ apoptosis through intracellular and extracellular apoptotic pathways, was proved to be the most cytotoxic among them (Zeng et al., 2020).

Zebrafish (*Danio rerio*), also known as a kind of tropical freshwater fish with ornamental value, is an important vertebrate model organism in scientific research, especially in studying the regenerative capacity of organisms (Goldshmit et al., 2012). Zebrafish and humans share up to 87% significant genome sequence similarity. It can overcome the limitations of cell-based *in vitro* assays and perform high-throughput *in vitro* screening (Bauer et al., 2021). Zebrafish are increasingly used in toxicity and targeted drug-related studies and have proved to be an essential vertebrate model for studying biological processes and functions (Barros et al., 2008; Ali et al., 2011; He et al., 2013).

This study aimed to investigate the potential hepatotoxicity of *Rhizoma Paridis* and its possible mechanism. To this end, we extracted *Rhizoma Paridis* by using petroleum ether, dichloromethane, ethyl acetate, n-butanol, and water, respectively. We examined the dose-toxicity curves of these five extracts by using zebrafish larvae. The relevant physicochemical indicators, including ALT/AST, hematoxylin-eosin staining, and acridine orange staining, were conducted to evaluate the liver damage of different extracts to zebrafish larvae. The chemical composition contained in extracts of *Rhizoma Paridis* was also analyzed by UHPLC-Q-Exactive Orbitrap MS. Modern lipidomics technology based on mass spectrometry has been used in this study to provide significant insights into the metabolism and alterations in lipids through the identification and qualitative analysis of individual lipid species. Meanwhile, lipid uptake, transport, metabolism, and inflammation-related genes were examined separately by quantitative real-time PCR (qPCR) in zebrafish larvae after exposure.

## Materials and Methods

### Chemicals and Materials

The HPLC grade methanol, acetonitrile, and water (Optima™ LC/MS grade) were purchased from Fisher Chemical, United States of America. Formic acid (HPLC grade) was purchased from CNW, Germany. 2-propanol (HPLC grade) was purchased from Merck (Darmstadt, Germany). MTBE (HPLC grade) was purchased from Adamas-beta. Ammonium acetate (HPLC grade) was purchased from Sigma-Aldrich (St. Louis, MO, United States). Petroleum ether, dichloromethane, ethyl acetate, n-butanol (analytical grade) were purchased from Sinopharm Chemical Reagent Co., Ltd (Shanghai, China). Ultrapure water was prepared using a Milli-Q water purification system (Millipore Corp. Billerica, MA, United States). Analytical grade ethanol and methanol were purchased from Beijing Chemical Works (Beijing, China).

### Preparation of *Rhizoma Paridis*

The dried roots of *Rhizoma Paridis* were collected from Maoshan town, Luquan country, Yunnan province, in October 2019. Professor Liu Chunsheng, who works at Beijing University of Chinese Medicine and is the head of the department of Chinese Medicine Identification, identified and checked the quality of these roots.

### Extraction and Fractionation

*Rhizoma Paridis* was powdered and successively extracted with five different solvents, petroleum ether, dichloromethane, ethyl acetate, water-saturated n-butanol, and water*.* The specific extraction method was as follows: The *Rhizoma Paridis* powder was wrapped with filter paper and placed in a Soxhlet extractor with solvent and extracted by heated reflux extraction within 2 h, followed by cooling and filtration. A rotary evaporator was used to recover the solvent, after which the remaining mixture was lyophilized into powder and stored at −4°C for further study. The extraction rates of ethyl acetate extract and dichloromethane extract of *Rhizoma Paridis* were 2.51 and 1.14%. The main components of the ethyl acetate extracts were analyzed using UPLC-Q-Orbitrap MS methods, which were described in detail in Supplementary Table S1.

### Animals and Experimental Design

#### Zebrafish Larvae Maintenance

Zebrafish wild-type (AB) were maintained in Farming System (ESEN, Beijing, China) in Beijing Key Lab for Quality Evaluation of Chinese Materia Medica, with a 14:10-h light/dark cycle and 28.5 °C water temperature. Fish were fed with brine shrimp three times a day. The male and female zebrafish were allowed to mate naturally every morning at 7 a.m. And healthy eggs were collected and cultured in embryo water (5.4 mmol/L KCl, 0.137 mol/L NaCl, 0.25 mmol/L Na_2_HPO_4_, 0.44 mmol/L K_2_HPO_4_, 1.3 mmol/L CaCl_2_, 1.0 mmol/L MgSO_4_, 4.2 mmol/L NAHCO3). Zebrafish embryos can take nutrients from the yolk sac before 7dpf. Healthy 4dpf larvae were screened by microscope (Zeiss, Germany) for further experiments.

#### Treatment of Zebrafish Larvae

Randomly selected 4dpf zebrafish larvae were placed in 12-well plates with 20 fish per well and exposed to different concentrations of *Rhizoma Paridis* extracts for 24 h, including petroleum ether extracts (PEE), dichloromethane extracts (DCME), ethyl acetate extracts (AcOEtE), water-saturated n-butanol extracts (WSBE) and water extracts (WE) of *Rhizoma Paridis* (dissolved in 0.5% dimethyl sulfoxide (DMSO)). Each extract contained different concentrations (containing both safe and lethal doses), and each concentration was repeated in three wells. Meanwhile, normal zebrafish culture water was used as a negative control group. During the exposure period, the number of dead zebrafish larvae in each experimental group was counted every 6 h, and the dead bodies were promptly removed. The entire experiment was carried out at a constant temperature of 28°C and repeated three times in parallel. At the end of the experiment, the number of surviving zebrafish larvae in the different treatment groups was counted to calculate the mortality rate of each group.

Then, the 4dpf zebrafish larvae were given the maximum non-lethal dose concentration of DCME and AcOEtE. 20 samples per well and six wells in parallel for each group, the blank group was given zebrafish embryo water. 24 h after administration, samples were collected and washed 3 times with embryo culture water and deionized water, respectively. Three wells of each group were fixed in 4% paraformaldehyde, and the remaining samples were stored at -80 °C for further study.

### Biochemical and Histological Analysis

#### Determination of Biochemical Parameters in Zebrafish Larvae

At 24 h postexposure, 80 zebrafish larvae were collected from each group, and a certain amount of phosphate-buffered solution (PBS, pH 7.4) was added. The specimens were homogenized by a homogenizer. Afterward, the specimens were centrifuged for about 20 min (2,000 rpm). The supernatant was carefully collected. Aspartate transaminase (AST) and alanine transaminase (ALT) levels were determined by using diagnostic kits according to the manufacturer’s protocols (Jiangsu Yutong Biotechnology Institute, Jiangsu, China). The activity of ALT and AST were determined at 450 nm after adding stop solution.

#### Histopathology Analysis

After 24 h exposure, zebrafish larvae were fixed with 4% paraformaldehyde for at least 24 h. For hematoxylin and eosin (H&E) staining analysis, zebrafish larvae were dehydrated using ethanol in ascending order and placed in xylene to make them gradually transparent, followed by paraffin embedding. Then 4 μm thick slides were prepared for H&E staining. For acridine orange staining, the embryos were washed 3 times with water and PBS at the end of exposing, and 1 ml of the acridine orange staining solution was added to each well and stained at 28°C for 20 min. The results were observed under a transmission electron microscope (ZEISS, German).

### Sample Preparation for Lipidomics Analysis

Zebrafish larvae samples (50 mg) were placed in 2 ml Eppendorf tubes, and 280 μL of extraction solution (methanol: water, 2:5), 400 μL of methyl-tert-butyl ether (MTBE) along with a 6 mm diameter grinding bead were added to the tube. Then, ground for 6 min (−10°C, 50 Hz) using a frozen tissue grinder. After that, the samples were extracted by low-temperature ultrasound for 30 min (5°C, 40 kHz), followed by 30 min of standing (−20°C). Then, samples were centrifuged at 13,000 g for 15 min at 4°C, and 350 μL of supernatant was added into the EP tube, followed by blow-drying with nitrogen gas. 100 μL of extraction solution (isopropanol: acetonitrile, 1:1) was added and re-solubilized, vortexed for 30 s, and low-temperature ultrasonic extraction for 5min (5 °C, 40 KHz). The extracts were centrifuged at high speed for 10 min (13,000g, 4°C), and the supernatant was pipetted into the injection vial with an internal cannula for analysis. In addition, 20 μL of supernatant was mixed for each sample as the quality control sample.

A Q-Exactive HF-X mass spectrometer (Thermo Scientific high-resolution) was coupled in tandem with a UHPLC system with a DuoSpray ion source operating in positive electrospray (ESI+) and negative electrospray (ESI-) modes (AB Sciex, Foster City, CA). After every 5–15 samples, a QC sample was inserted, and an automatic mass calibration was performed to examine the stability of the entire essay. An Accucore C30 column (100 mm × 2.1 mm i. d. 2.6 μm; Thermo) was used for the separation of lipids. The temperature of the column was maintained at 40 °C. Mobile phase A was 50% acetonitrile in water (containing 0.1% formic acid, 10 mmol/L ammonium acetate), and mobile phase B was acetonitrile: isopropanol: water (10:88:2, v/v/v, containing 0.02% formic acid, 2 mmol/L ammonium acetate). The flow rate was 0.4 ml/min, and the gradient analysis was performed with a gradient starting from 35% mobile phase B, 60% B (4 min), 85% B (12 min), 100% B (15–17 min), and 35% B (18–20 min). After UHPLC separation, samples were analyzed by Q ExactiveTM HF-X for mass spectrometry with the following instrument parameter settings: ion mode, positive and negative; MS1 scan range, 200-2000; Irospray voltage Floating (ESI+, V), 3.0 kV; ionspray Voltage Floating (ESI-, V), −3.0 kV; heater temperature, 370°C; sheath gas flow rate, 60 psi; aux gas flow rate, 20 psi; capillary temperature, 350°C; Normalized collision energy, 20-40-60 (V). Details are described in Supplementary Table S2.

### Quantitative Real-Time PCR Analysis of Related Gene Expression

Total RNA was extracted from collected samples using TianGen RNA Extraction Kit (Takara, Tokyo, Japan) following manufacturer instructions. The isolated total RNA from the different groups was converted into complementary DNA (cDNA) using the FastKing RT Kit (Tiangen Biotech Co., Ltd., Beijing, China). Sequences of primers used in the qPCR were designed with primer-blast (https://ncbi.nlm.nih.gov/tools/primer-blast/) and synthesized by the Biomed gene technology co. LTD. (Beijing, China). (Supplementary Table S3). Quantitative real-time PCR was performed using TransStart Top Green qPCR SuperMix (TransGen Biotech, Beijing, China) with the ABI 7500 Real-Time PCR System (Applied Biosystems, Foster City, CA, United States). Relative mRNA expression levels were determined using the following thermal cycling conditions: 95°C for 3 min, followed by 40 cycles of 60°C for 20 s, 60°C, 9°C for 15 s, and 6°C for 10 s, and 9°C for 15 s. The relative quantification was then calculated by the expression 2^−∆∆Ct^. All data were statistically analyzed as the fold-change between the exposed groups and the control. The experiment was conducted in triplicate.

### Statistical Analysis

The components of the samples were separated by chromatography and continuously scanned into the mass spectrometer, and the mass spectrometer was continuously scanned for data acquisition. The raw data were imported into LipidsearchTM 4.1 (Thermo Fisher, United States) for baseline filtering, peak identification, integration, retention time correction, and peak alignment to obtain a data matrix containing retention time, mass-to-charge ratio, and peak intensity information. The MS and MS/MS mass spectra were matched with the metabolic database with the MS mass error set to less than 10 ppm, and the metabolites were identified based on the secondary mass spectra matching score. The main parameters are as follows: precursor tolerance: 5ppm, product tolerance: 5ppm, product icon threshold: 1%. For the LipidSearch extracted data, the lipid molecules in the missing value 20% group were removed, and the missing values were filled with the minimum value, QC verified with RSD ≤30% and the total peak area of the data was normalized. Subsequently, model construction and discrimination of multivariate statistics were performed using the ropls R package, which included unsupervised principal component analysis (PCA) and orthogonal partial least squares discriminant analysis (OPLS-DA). Among them, PCA analysis uses unite-variance to scale transform the data, and (O) PLSDA uses Pareto to scale transform the data. The values of *R* ^2^ and Q^2^ were calculated to assess how good the quality of both models was. Volcano plots were plotted using the R package ggplot2. One-way statistical analyses included Student’s t-test and mutation multiple analysis. Volcano maps were drawn using r software for hierarchical clustering and correlation analysis. Differences between groups (*p*-values) were assessed using Graphpad Prism 8.0 software. Differences in data were first determined using normality and chi-square tests, followed by one-way ANOVA analysis and Dunnett’s *t*-test. *p ≤ 0.05 and * *p ≤ 0.01 were statistically significant for the AcOEtE group compared to the DMSO group.

## Results

### Toxicity of Different Solvent Extracts of *Rhizoma Paridis*

**FIGURE 1:**
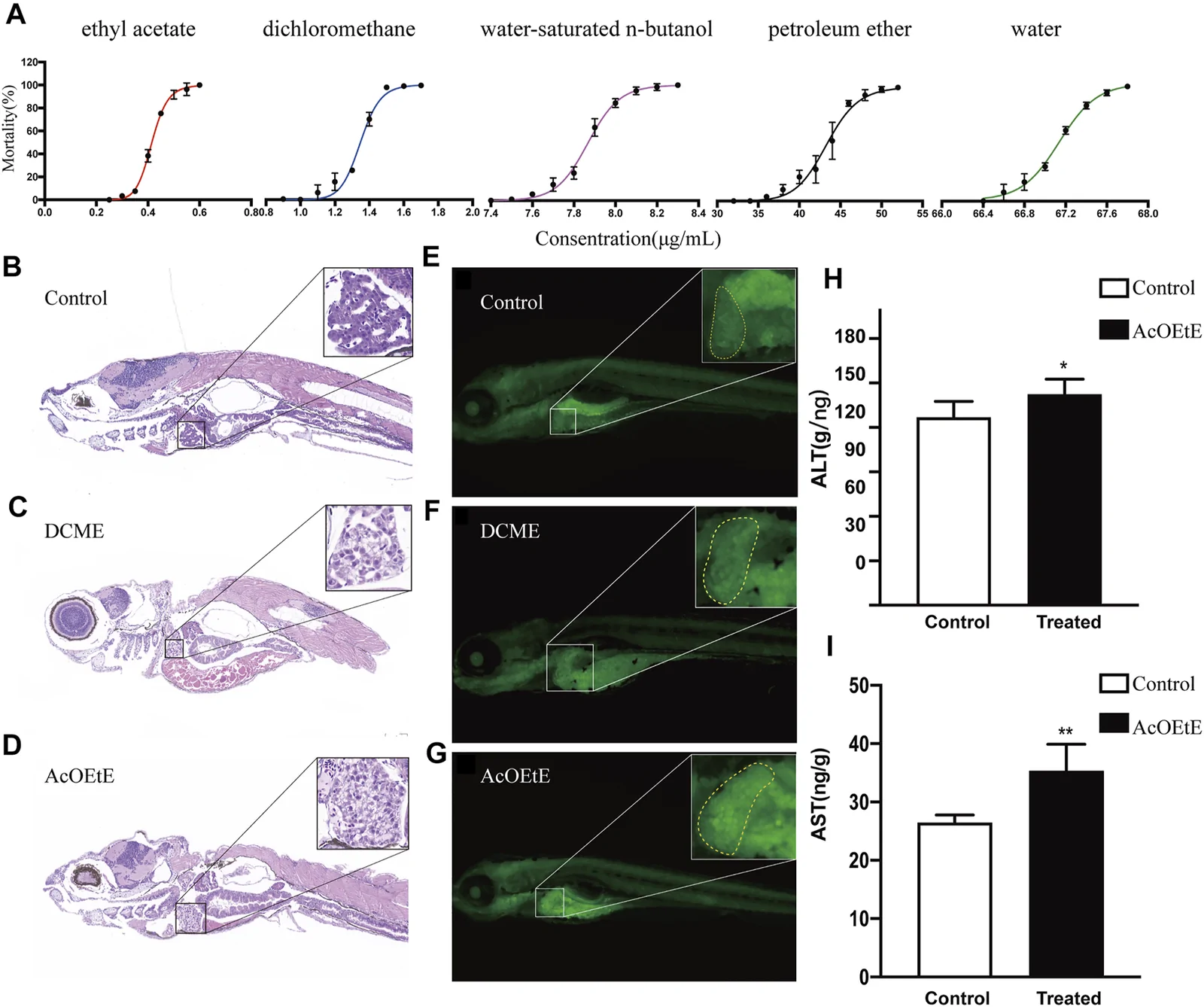
Concentration-mortality curves, tissue sections (×400), and biochemical indicator analysis **(A)** Concentration-mortality curves of five solvent extracts of *Rhizoma Paridis*, including petroleum ether extracts (PEE), dichloromethane extracts (DCME), ethyl acetate extracts (AcOEtE), water-saturated n-butanol extracts (WSBE), and water extracts (WE) **(B-D)** H&E staining of zebrafish larvae sections from the control, DCME, and AcOEtE groups **(E–G)** Acridine orange staining of zebrafish larvae from the control, DCME, and AcOEtE groups **(H–I)** The levels of ALT and AST of control and AcOEtE groups. Data are expressed as mean ± SD. **p < 0.01 compared with the control group, *p < 0.05 compared with control group.

Previous studies have shown that the aqueous extracts of *Rhizoma Paridis* exhibited hepatotoxicity to zebrafish and SD rats and cytotoxicity to L02 cells (Zhao et al., 2020). However, there have been no studies on the differences in toxicity of extracts from different solvents on *Rhizoma Paridis*. Our study compared the toxicity of different extracts of *Rhizoma Paridis* on zebrafish larvae. The “dose-toxicity curve” of different extracts is shown in Figure 1A. The results showed that the different extracts of *Rhizoma Paridis* exhibited a wide variation in toxicity to zebrafish larvae. As shown in Figure 1A, the ethyl acetate extracts of *Rhizoma Paridis* (AcOEtE) were the most toxic fraction, followed by dichloromethane extracts (DCME), petroleum ether extracts (PEE), water-saturated n-butanol extracts (WSBE), and water extracts (WE).

Afterward, AcOEtE and DCME were selected as the more toxic fraction to investigate whether they are hepatotoxic and potentially induce hepatic fibrosis. The administered concentrations are their respective maximum non-lethal concentrations, for AcOEtE was 0.3 μg/ml, and DCME was 1 μg/ml. Randomly selected 4dpf zebrafish larvae were placed in 12-well plates with 20 fish per well and exposed to 0.3 μg/ml AcOEtE and 1 μg/ml DCME for 24 h respectively. Pathological changes were observed in zebrafish larvae 24 h after the administration of AcOEtE and DCME.

### Histological and Biochemical Analysis

The results of H&E (Figures 1B–D) showed that AcOEtE and DCME had caused varying degrees of liver damage to zebrafish larvae, with inflammatory cell infiltration and mild vacuolated lipid droplets in both groups. In contrast, AcOEtE was significantly more hepatotoxic to zebrafish larvae than DCME, and significant histopathological changes could be observed in the AcOEtE group, including significant hepatic steatosis, partial nuclear fragmentation, and the appearance of hepatic fibrous nodules. Acridine orange staining showed that both AcOEtE and DCME promoted apoptosis in the hepatocytes of zebrafish larvae to some extent (Figure 1E–G). Meanwhile, the results of ALT/AST (Figure 1H) after 24 h administration of AcOEtE to zebrafish larvae showed that both ALT/AST significantly increased in the treated group compared to the control group.

### Identification of Chemical Compounds in the AcOEtE

Previous studies have been conducted on the chemical composition of *Rhizoma Paridis* and saponins, which are usually considered the main components. In this study, we used UPLC and high-resolution Q-Exactive Orbitrap MS to identify the constituents of AcOEtE. Based on the high-resolution parent ions and their characteristic ions, we have identified 98 relevant components, including Polyphyllin I, Polyphyllin VI, Polyphyllin E, Dioscin, Diosgenin, Protodioscin, Gracillin, etc. The chemical compositions are shown in Supplementary Table S4.

### Lipid Analysis and Multivariate Analysis of UHPLC-Q Exactive HF-X Mass Spectrometer Data

Lipidomics, derived from metabolomics, emerged in 2003 and has rapidly evolved to focus on the qualitative and quantitative screening of metabolites in biological samples, with two main analytical approaches: 1) the non-targeted and 2) the targeted approach (Lee and Yokomizo, 2018). This study performed lipidomic analysis on zebrafish larvae treated with AcOEtE and control groups based on the UHPLC-Q Exactive HF-X-MS technique, respectively. Supplementary Figure S1 shows an example of the total ion chromatogram (TIC) of the QC group in positive and negative ion mode, which showed good separation.

**FIGURE 2:**
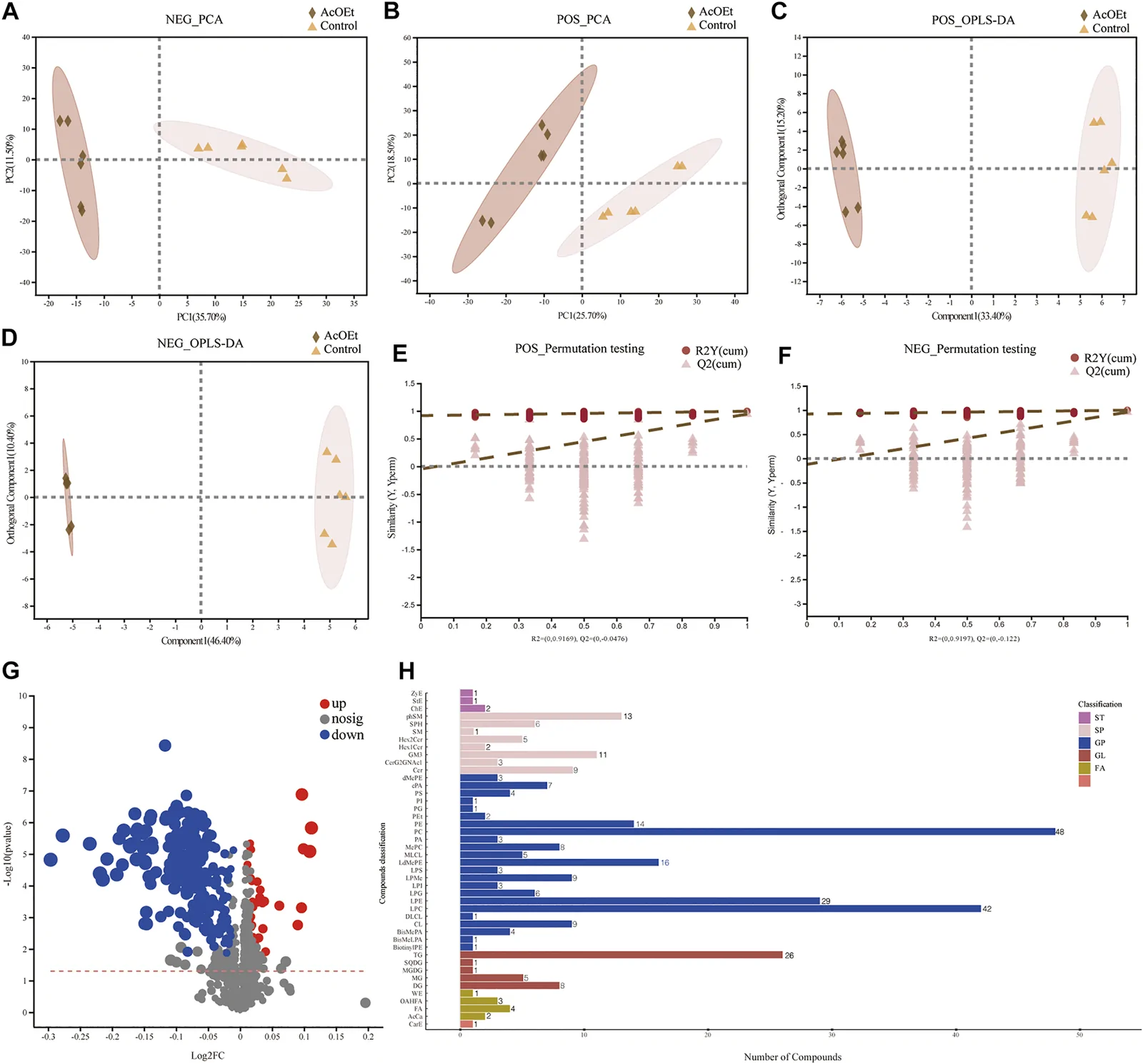
Score plot of PCA and OPLS-DA models of zebrafish larvae **(A, B)** Scores plot of PCA model, obtained from AcOEtE group and control group (NEG and POS) **(C,D)** Scores plot of OPLS-DA model, obtained from AcOEtE group and control group (NEG and POS) **(E,F)** The score of the OPLS-DA model, obtained from the AcOEtE group and control group (NEG and POS). The *R* ^2^ factor estimates a good fit, while the Q^2^ coefficient determines the predictive value of the created model, referring to the percentage of correctly classified samples using cross-validation. Two hundred permutations were performed, and the resulting *R* ^2^ and Q^2^ values were plotted (Red circle): *R* ^2^ (Pink triangle): Q^2^. The two dashed lines represent the regression lines of *R* ^2^ and Q^2^, respectively **(G)** volcano plot of Model-Control, the blue points represent the down-regulated lipids, red points represent the up-regulated lipids (VIP >1.0, *p*-value < 0.05) **(H)** The number of detected lipids was based on a non-targeted lipidomics strategy between the AcOEtE and control groups. The horizontal axis represents the number of lipids in the subclass, and the vertical axis represents the subclasses of lipids.

Principal Component Analysis (PCA) and Orthogonal Projection of Latent Structure Discriminant Analysis (OPLS-DA) are powerful statistical modeling tools that provide insight into the separation between experimental groups based on the results from the MS analysis instrument and determine whether an experimental sample is anomalous based on its dispersion trend (Worley and Powers, 2016). As can be seen from Figures 2A–D, the QC samples were tightly aggregated in ESI+ and ESI- modes, indicating that the instrument was in good operating condition. Both PCA and OPLS-DA results completely distinguish the AcOEtE group from the control group, indicating that endogenous metabolite levels in the treated group have changed significantly compared to the control group.

The results showed that R^2^X (cum), R^2^Y (cum), and Q^2^ of the OPLS-DA model for the cationic model were 0.486, 0.996, and 0.944, respectively, while R^2^X (cum), R^2^Y (cum), and Q^2^ of the anionic model were 0.568, 0.998 and 0.958, respectively (Figures 2C,D). Q^2^ > 0.5 indicated that the model’s predictive ability was good, but only using Q^2^ is still not enough to prove the model’s reliability, and the replacement test-permutation test is also commonly used to judge the model. A 200-time permutation test was performed to validate the OPLS-DA model. Compared to the original points, the Q^2^ and *R* ^2^ values on the left side are lower, while the intercept of the regression line at the Q2 point with the vertical axis (left side) is less than 0.05 in the envelope test (Figures 2E,F). This indicates that the model is valid and can make accurate predictions for the samples in the experiment.

Each high-resolution mass spectrometry peak extracted by compound Discoverer TM was initially screened and classified into lipid subspecies based on their different cleavage patterns and further confirmed by comparing precise mass determination and the given molecular formula with Lipidsearch (Thermo Fisher, United States) on the lipid database for further confirmation. The International Lipid Classification and Nomenclature Committee classify lipid compounds into eight types, each containing different subclasses, and each subclass can be further divided into different molecular species. We then screened the metabolites for those that were altered in zebrafish larvae after administration. The significance of the variables in the projection (VIP >1) values generated in OPLS-DA was applied in the positive and negative ion model, respectively, resulting in 325 differential metabolites were screened out (Figure 2G), covering five lipid categories and 43 lipid subclasses, with phosphatidylcholines (PCs), PEs, and triglycerides (TGs) were the most abundant (Figure 2H).

**FIGURE 3:**
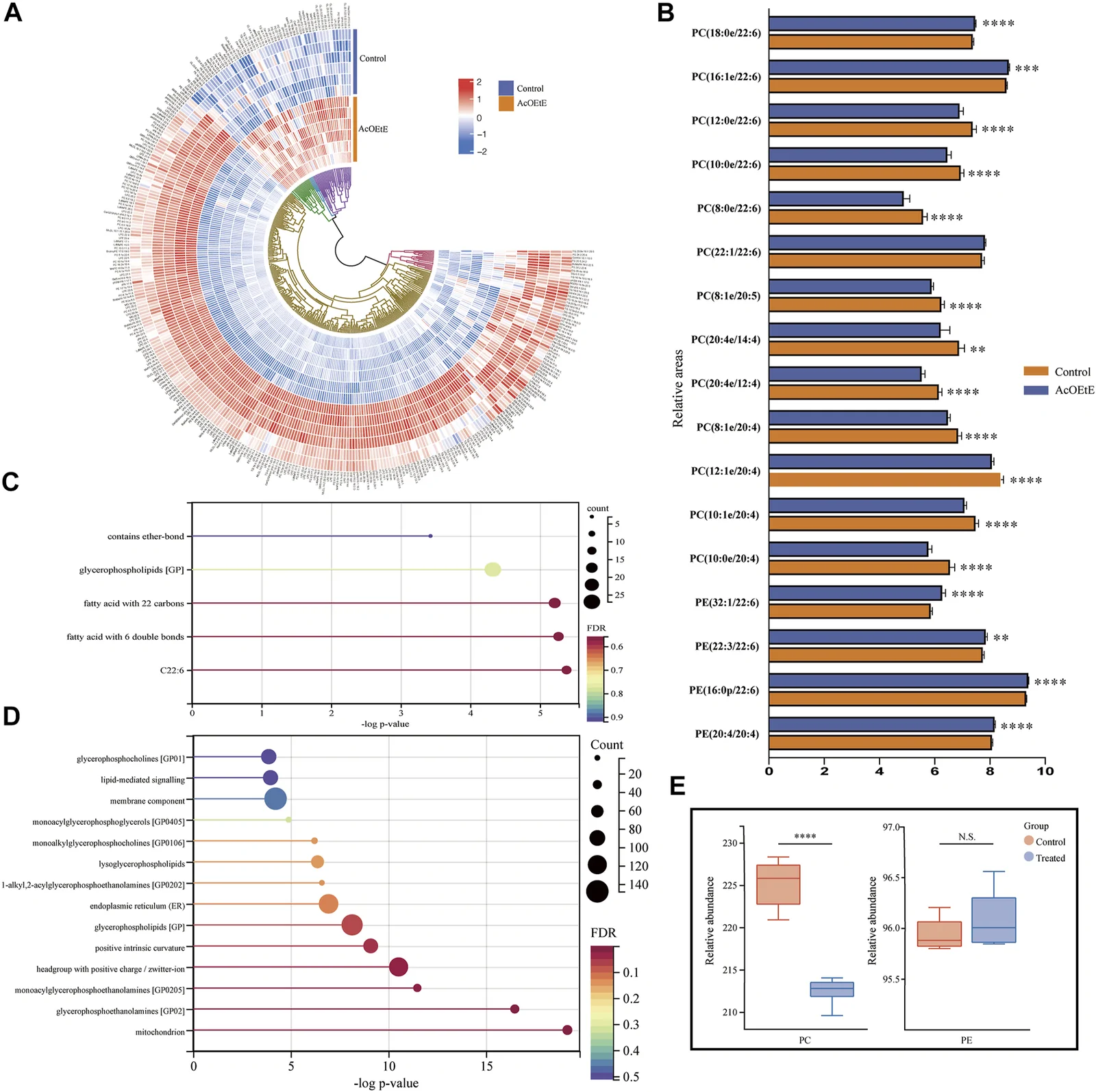
Analysis of abnormal lipid regulation by AcOEtE in zebrafish larvae **(A)** Heatmap of 325 lipids in the control and AcOEtE groups. The red and blue colors indicate the increased and decreased levels, respectively **(B)** Relative intensity analysis of subclasses containing eicosatetraenoic acid or docosahexaenoic acid between the control and AcOEtE groups **(C)** LION-term enrichment analysis of all up-regulated lipids in the AcOEtE group compared to the control group **(D)** LION-term enrichment analysis of all down-regulated lipids in the AcOEtE group **(E)** PCs and PEs in control and AcOEtE groups showed a rise in total concentration. *p < 0.05, **p < 0.01 compared with the control group.

We analyzed the lipid data using clustered heat maps to understand further the differences in lipid metabolism between the AcOEtE and control groups, as shown in Figure 3A. The heatmap visualizes the relative increase (red) or decrease (blue) of lipids in each group of samples. 269 lipids were significantly downregulated, and 56 lipids were significantly upregulated in the AcOEtE group compared to the Control group.

Hierarchical clustering analysis strongly emphasized the enrichment of CL and PE in glycerophospholipid, sphingomyelin (Sph), and Cer in sphingolipids, and sterol lipid. Specifically, among the sphingolipids, six out of nine Cers, three out of five Hex2Cers, and two out of six Sphs showed significant upregulation in the AcOEtE group. Glycerophospholipids were identified in a total of 23 subclasses, and five of these subclasses were significantly upregulated in the AcOEtE group, including CLs, PEs, phosphatidylglycerols (PGs), LPIs, and dimethyl-phosphatidylethanolamines (dMePEs).

Then, we performed a further generalization study with the Lipid Ontology website (http://www.lipidontology.com/) on 269 down-regulated lipids and 59 up-regulated lipids, which were significantly increased by AcOEtE exposure. The results showed that a total of 14 lipids containing docosahexaenoic acid, 15 lipids containing fatty acids with six double bonds, 18 lipids containing fatty acids with 22 carbon atoms, 27 glycerophospholipids, and three lipids containing ether bonds were among the 59 lipids up-regulated after AcOEtE exposure. These five physicochemical properties occupy the top five positions in terms of contribution to lipid upregulation, as shown in Figure 3C. Meanwhile, we can see that the 269 lipids down-regulated are mainly related to mitochondria, endoplasmic reticulum, cell membrane components, positive intrinsic curvature of the cell membrane, head group of lipids with positive charge/zwitterion, and lipid-mediated signaling, etc. As shown in Figure 3D.

PCs and PEs were the most abundant phospholipids in mammalian cells and showed significant changes in various diseases. Long-chain polyunsaturated fatty acids (LC-PUFA) were defined as polyunsaturated fatty acids with more than 20 carbon atoms and two or more double bindings (Inam AKM and Suzauddula, 2017). Eicosapentaenoic acid (EPA, C20:5n-3), arachidonic acid (AA, C20:4n-6) and docosahexaenoic Acid (DHA, C22:6 n-3) were considered as the most important LC-PUFA. We investigated the changes of PCs and PEs between the AcOEtE and control groups and focused on the changed subclasses that contained four or more double bonds. The results of our research suggested that among the 48 significantly altered PC subclasses detected in the AcOEtE group, 16 subclasses are composed of unsaturated fatty acids that contain more than 20 carbon atoms and two or more double bonds. Among the 48 significantly changed PC subclasses, four were significantly upregulated, accounting for 8.3%. It is worth mentioning that all the upregulated subclasses were LC-PUFA, specifically three subclasses were docosahexaenoic acid and one docosatetraenoic acid. Of the 14 significantly altered PE subclasses, five subclasses are composed of unsaturated fatty acids that contain more than 20 carbon atoms and two or more double bonds. Compared with the control group, 11 PE subclasses of the AcOEtE group showed significant upregulation, accounting for 78.6%, of which five contained docosahexaenoic acid and one contained docosahexaenoic acid, as is shown in Figure 3B. The PC/PE ratio is often used clinically to detect inflammatory conditions in the organism. Compared to the control group, the PCs mainly were downregulated in the administered group, while PEs were upregulated overall in the AcOEtE group (except for the two short-chain lipids), which led to a substantial downregulation of the ratio (Figure 3E).

In addition, all four sterol lipids subclasses detected in the AcOEtE group, including ChE (24:5), ChE (24:6), StE (24:7), and ZyE (24:5), were all long-chain sterols and upregulated, which may be related to signaling pathway conduction.

**FIGURE 4:**
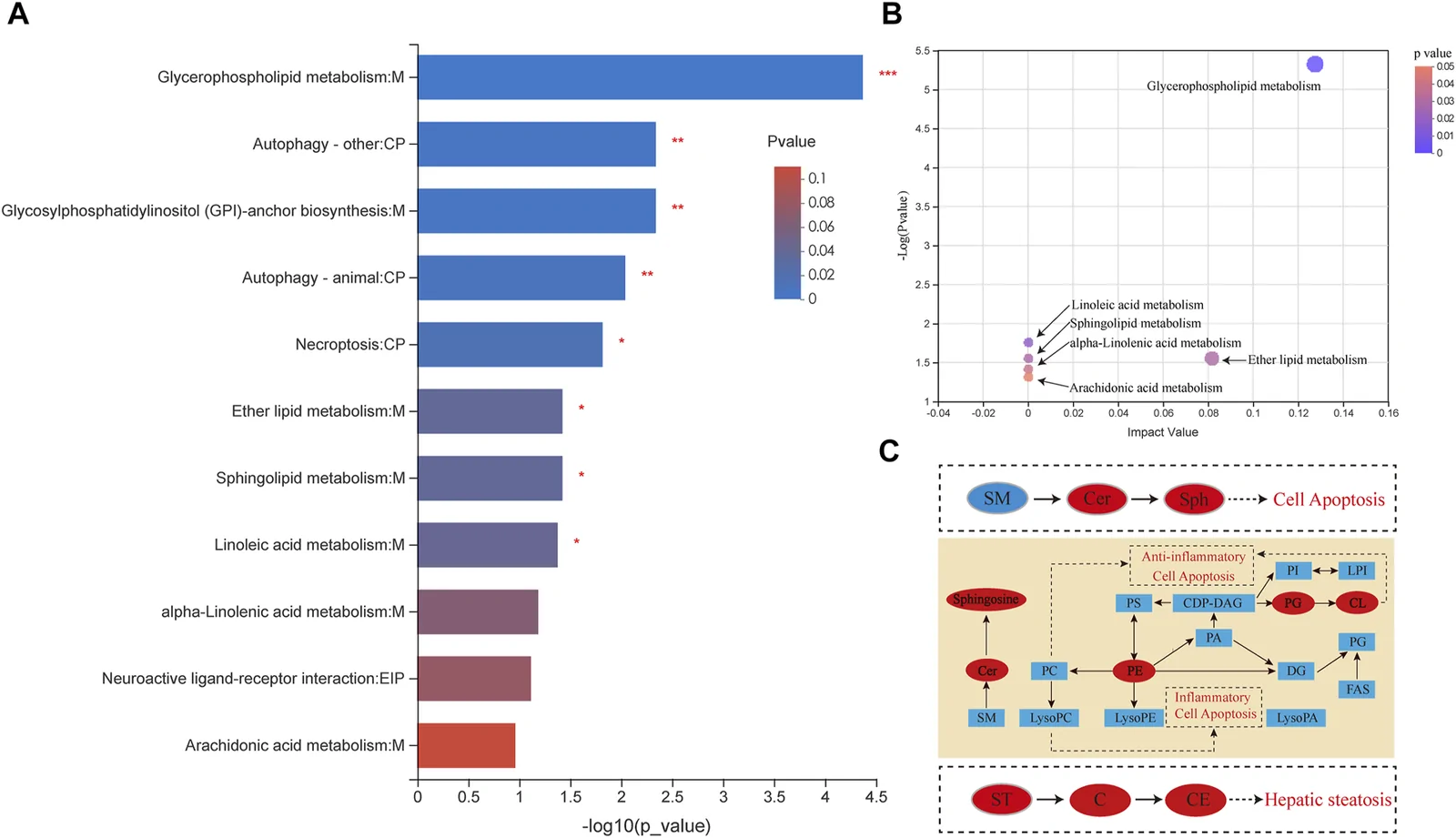
Different lipid species are associated with metabolism **(A)** Pathway enrichment analysis of lipid metabolites by KEGG. Results include α-linolenic and linoleic acid metabolism, arachidonic acid metabolism, glycerolipid metabolism, fatty acid extension in mitochondria, fatty acid biosynthesis, fatty acid metabolism, steroid biosynthesis, and bile acid biosynthesis (*p < 0.05, **p < 0.01 compared with the control group) **(B)** Pathway topology enrichment analysis of lipid metabolites by KEGG **(C)** Network of changes in potential biomarkers of the AcOEtE group compared to the Control group. Red, upregulated biomarkers; blue, downregulated biomarkers.

To determine the pathways by which AcOEtE affects lipid metabolism in zebrafish larvae, scipy (Python) software was used for analysis. *p*-value was analyzed and resulted in 39 metabolites mapped to the KEGG metabolic pathway for overexpression and pathway topology analysis. Different metabolite species were analyzed using Scipy. *p*-value corrected less than 0.05, and finally, 11 lipid metabolic pathways were found to have occurred a game (Figure 4A). By the relative positions of the compounds in the pathways, their pathway composite importance scores were calculated with a total score of 1. As shown in Figure 4A. The figure shows that glycerophospholipid metabolism, ether lipid metabolism, linoleic acid metabolism, and sphingolipid metabolism had the highest impact factors.

The results of KEGG topology statistics are shown in Figure 4B, where each bubble represents a KEGG pathway, and the vertical axis indicates the enrichment significance of metabolite involvement in the pathway-log10 (*p*-value); the bubble size represents the Impact Value; the more significant the bubble, the greater the importance of the pathway. Five pathways can be found in the figure, including map00564 (Glycerophospholipid metabolism), map00591 (Linoleic acid metabolism), map00590 (Sphingolipid metabolism), map00592 (alpha-Linolenic acid metabolism), and map00565 (Ether lipid metabolism).

### Effect of Critical Lipid-Relating Genes by AcOEtE

**FIGURE 5:**
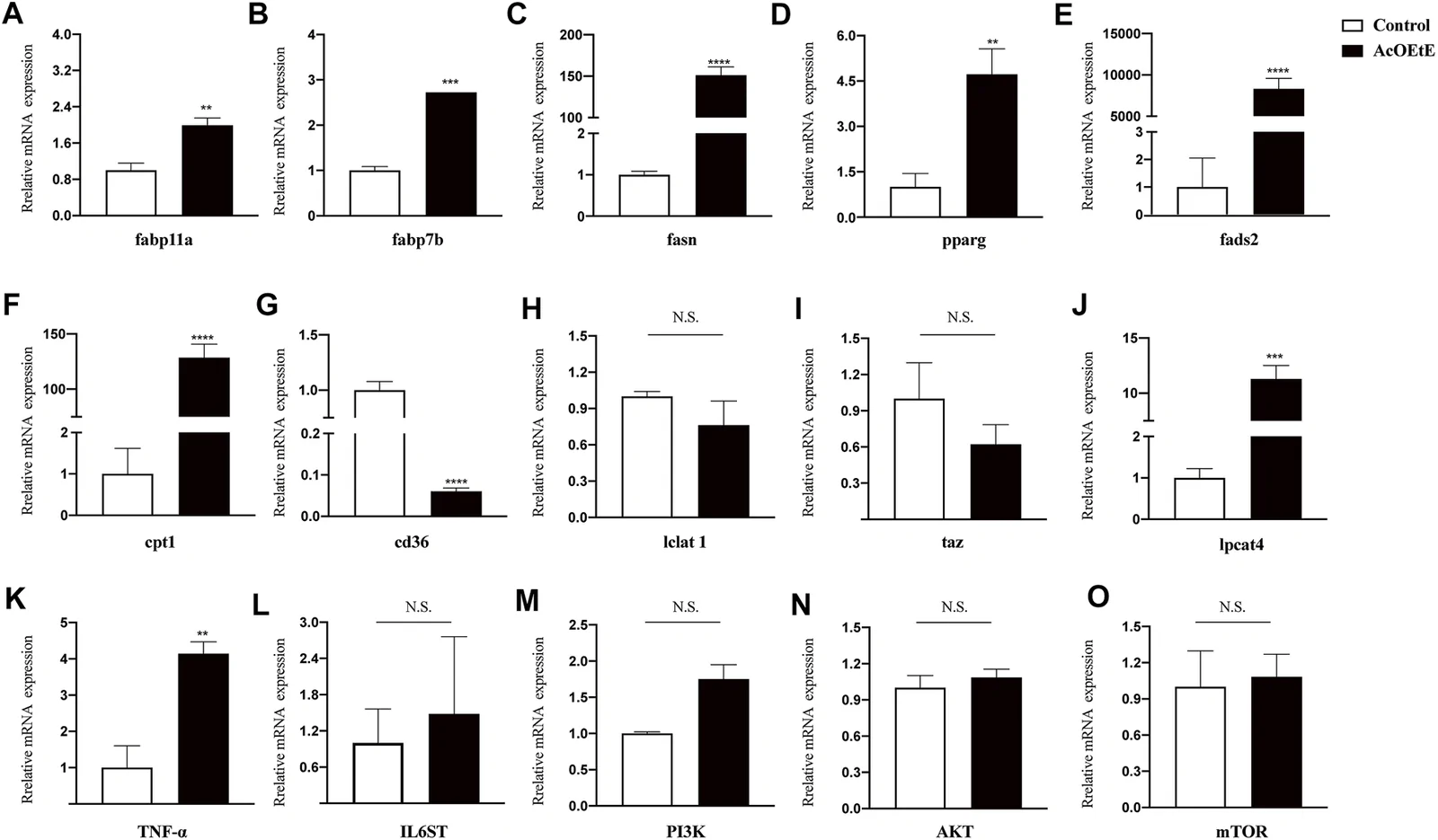
Gene expressions after AcOEtE administration to zebrafish larvae were detected by qPCR. including **(A)** fabp11a **(B)** fabp7b **(C)** fasn **(D)** pparg **(E)** fads2 **(F)** cpt1 **(G)** cd36 **(H)** lclat1 **(I)** taz **(J)** lpcat4 **(K)** TNF-α **(L)** IL6ST **(M)** PI3K **(N)** AKT and **(O)** mTOR. All values are expressed as the mean ± SD of three or more independent experiments. ***p < 0.001; **p < 0.01; *p < 0.05.

To determine the underlying mechanism of the abnormal lipid metabolism, the transcription levels of genes that participated in lipid metabolism were further assessed by qPCR (Figure 5). Our results indicated that AcOEtE affected genes related to lipid synthesis, metabolism, and transport in zebrafish larvae, such as fabp11a, fabp7b, fasn, pparg, fads2, cpt1, cd36, and enzyme activities related to mitochondrial function were also activated, such as lclat1, Lpcat4, and Taz. Genes related to inflammation were also upregulated, such as TNF-α, IL6ST. As found in our lipidomics results, these genes are involved in the glycerophospholipid metabolic pathway, α-linolenic acid metabolic pathway. Meanwhile, we found a tendency for the PI3K-AKT-mTOR pathway to be upregulated, although it did not show significance, as shown in Figure 5M–O.

## Discussion

In this study, we investigated the dose-toxicity relationship of different extracts of *Rhizoma Paridis* on zebrafish larvae. We studied the induction of hepatic fibrosis after administration of AcOEtE (ethyl acetate extracts of *Rhizoma Paridis*) and DCME (dichloromethane extracts of *Rhizoma Paridis*) by pathological morphological observation and physicochemical index analysis.

Then, we investigated the effect of AcOEtE on lipid homeostasis in zebrafish larvae by using lipidomics techniques to search for biomarkers that may play a key role in inducing hepatic fibrosis. To the best of our knowledge, this is the first study of hepatotoxicity and hepatic fibrosis in a zebrafish larvae model using lipidomic techniques, which may provide a good reference for future hepatotoxicity studies and clinical applications of *Rhizoma Paridis*.

Compared with the control group, after ingestion of a specific dose of AcOEtE, the lipid homeostasis in zebrafish larvae was disrupted and exhibited pathological fibrosis changes. We examined the mRNA levels of pro-inflammatory factors IL6ST and TNF-α in zebrafish larvae of the AcOEtE group, and both were upregulated. Genes related to fat synthesis and transport, such as the fatty acid-binding protein FABP family, were also detected. FABPs is a single molecular weight intracellular protein family, mainly involved in the transport and metabolism of fatty acids. We examined Fabp7b and Fabp11a, and both showed a significant upregulation trend in the AcOEtE groups. Upregulation of fabp11a and fabp7b genes leads to increased hepatic fatty acid uptake, affecting the subsequent lipid transport process and increasing hepatic lipid deposition and ultimately hepatic steatosis (Ahn et al., 2012; Yan et al., 2015). Fasn is an essential nuclear transcription factor that regulates hepatic lipid metabolism and is a crucial regulator of fatty acid metabolism, and its excessive upregulation leads to hepatic lipid disorders in zebrafish, which in turn causes hepatic lipid accumulation (Chicco and Sparagna, 2007; Knebel et al., 2018; Peng et al., 2018). There was a strong correlation between increased pparg expression and liver fat accumulation. In addition, pparg overexpression was accompanied by increased transcription of several inflammatory marker genes (Westerbacka et al., 2007; Yamazaki et al., 2011). Also upregulated was the fatty acid dehydrogenase 2 (fads2), a key enzyme in synthesizing polyunsaturated fatty acids.

### Changes in the Glycerophospholipid Metabolism Pathway

The results of lipidomic studies showed that zebrafish larvae underwent significant changes in the glycerophospholipid metabolic pathway after administration of AcOEtE, with the more significant changes being in PC and PE. There was a total of 48 PCs and 14 PEs were detected to be significantly altered in the AcOEtE group, of which 4 PC subclasses were upregulated (8.33%), and 11 PEs were upregulated (78.57%), suggesting that the homeostasis of PEs and PCs is disturbed in the glycerophospholipid metabolic pathway, and lead to changes in membrane lipid composition, which could affect the physical properties and functional integrity of membranes, leading to apoptosis, inflammation and hepatic fibrosis (Li et al., 2006; Wu et al., 2019). Puneet Puri et al. showed that total PCs were reduced in the livers of humans with Non-alcoholic fatty liver disease (NAFLD) and Non-alcoholic steatohepatitis (NASH) (Puri et al., 2007). Arendt et al. found that NAFLD and NASH patients had significantly lower PC/PE ratios in liver and erythrocyte membranes than healthy people (Owen and McIntyre, 1978). Charalampos et al. also found that the PC/PE ratio showed a negative correlation with ALT and AST and thus hypothesized that the PC/PE ratio correlated with the pathology of liver tissue (Papadopoulos et al., 2020).

Dimethyl-phosphatidyl ethanolamine (DMePE) is an intermediate in the sequential methylation of PE, and this metabolic pathway is one of the main metabolic pathways for the *de novo* synthesis of PC (Cole et al., 2012). In this study, the amount of PC was reduced in the AcOEtE group compared to the control group, while the amount of DMPE was significantly increased, possibly due to an impairment in this biosynthetic pathway.

Our experimental results also revealed that the Lysophosphatidylcholine (LPC) of the AcOEtE group showed a tendency to be down-regulated to some extent compared to the control group. A total of 42 isoforms were detected in LPC, which were all down-regulated in the AcOEtE group compared to the control group. LPCs are essential signaling molecules with multiple biological functions. LPC affects lipid metabolism throughout the liver and has been found to down-regulate genes involved in fatty acid oxidation and upregulate genes involved in cholesterol biosynthesis (Hollie et al., 2014). Tanaka et al. found that downregulation of serum LPC was significantly correlated with hepatic upregulation of LPcat1-4 and the pro-inflammatory cytokines TNF-α upregulated the expression of Lpcat2/4 mRNA levels in primary hepatocytes (Tanaka et al., 2012). Our experiment showed that the AcOEtE group had a higher level of Lpcat4 compared to the control group. Furthermore, it was also found that TNF-α was significantly upregulated, and IL6ST showed an upregulation trend in the AcOEtE group, indicating that the upregulated expression of Lpcat4 was associated with inflammatory cytokines.

Another possible reason for the overall downregulation of PCs in the AcOEtE group is the involvement of the alpha-linolenic acid metabolic pathway. PCs could be converted to alpha-linolenic acids, which could be further converted to stearidonic acids in the alpha-linolenic acid metabolic pathway. The KEGG pathway analysis of lipidomic results supports this hypothesis, as does the upregulation of activity of fads2 enzyme (an enzyme required for the conversion of PCs to α-linolenic acid) in the AcOEtE group.

CL plays an essential role in mitochondrial bioenergetic processes and is localized and synthesized in the inner mitochondrial membrane, where biosynthesis occurs (Paradies et al., 2019). Also, excess CL has been shown to have a detrimental effect on mitochondria. CL is closely associated with respiratory chain proteins and is therefore very sensitive to peroxidation, which may lead to the induction of an apoptotic cascade response, ultimately leading to programmed cell death (Petrosillo et al., 2001; Paradies et al., 2002; Paradies et al., 2014). To our Experimentally, CL species containing longer fatty acyl chains were found to be significantly increased in the AcOEtE group. We also examined the level of genes involved in mitochondrial activities inside the AMPK pathway. Carnitine palmitoyltransferase-1 (cpt1) is an enzyme responsible for transporting long-chain fatty acids for β-oxidation, regulating cellular differentiation and lipid metabolism (Zhang et al., 2014). Cpt1 is a rate-controlling enzyme for fatty acid β-oxidation, catalyzing the condensation of acyl-coenzyme A with levulinic acid to form acylcarnitine esters, which are subsequently transported to mitochondria for further catabolism (McGarry and Brown, 1997). In our experiments, we found that the activity of cpt1 was significantly upregulated in the AcOEtE group, which could explain the overactive mitochondrial function in the CL supersaturation state and the translocation and processing of long-chain fatty acids involved in the glycerophospholipid metabolic pathway.

Phosphatidylglycerol (PG) is a glycerophospholipid that is an intermediate product of the CDP-DG (CDP 1,2-diacyl-sn-glycerol) synthetic pathway and a precursor for the synthesis of CL (Kiyasu et al., 1963; Hostetler et al., 1971). The upregulation of PGs in the experimental results may be related to the over-activation of the synthetic pathway of CLs. Our results revealed that PG (18:1/20:5) content was upregulated in the AcOEtE group, and all nine subclasses detected in CL were upregulated. The upregulation did not occur in the five isoforms of MLCL. The activities of the Lclat1 gene and Taz gene were examined, and both were downregulated, indicating that the MLCL to CL conversion pathway was inhibited, possibly as an antiphase protective mechanism of the organism. Compared to the control group, LPG was not upregulated in the administered group, but the enzyme activity of LPG to PG was upregulated, which explained why the PG content was upregulated. Meanwhile, we examined the activity of the cd36 enzyme and found that cd36 underwent significant downregulation. Cd36 is a scavenger receptor that functions in the high-affinity tissue uptake of long-chain fatty acids (FA) and is involved in cellular fatty acids FA uptake. In the AMPK pathway, cd36 could directly promote the further conversion of fatty acids to Fatty Acyl-Coa, while there was a significant upregulation of the mitochondria-related enzyme cpt1, indicating that mitochondria-related functions were activated.

### Changes in the Sphingolipid Metabolism Pathway

Our results identified upregulation of Cer and sphingomyelin (Sph) in the AcOEtE group (Figure 3A), both of which are included in the sphingolipid metabolic pathway. Cers and ceramide-derived sphingolipids are structural components of cell membranes associated with oxidative stress and inflammation and may play a role in developing Hepatic fibrosis. Inflammation and an oversupply of saturated fatty acids stimulate the continuous synthesis of new Cers (Kasumov et al., 2015). Our results showed that significant upregulation of nine Cer subclasses occurred in the AcOEtE group compared to the control group, while qPCR results also indicated that AKT was suppressed in the AcOEtE group compared to the control group. The level of Cer was increased after the administration of AcOEtE, which may lead to apoptosis of hepatocytes.

As for Sph, present in animal cell membranes, it is usually composed of choline phosphate and Cer or PE head groups. In our study, some of the Sphs in the AcOEtE group were also upregulated. Despite its very low abundance, it is an essential structural component of the cell membrane (Quehenberger et al., 2010; Rodriguez-Cuenca et al., 2017). It was found that after 16°weeks of high fat and high cholesterol administration to mice, the Sph content in their liver increased significantly (Sanyal and Pacana, 2015). Cer, Sph, and sphingosine 1-phosphate (S1P) can be interconverted, a delicate balance known as the “sphingosine rheostat” (Musso et al., 2018). If Cer and Sph are increased, then apoptosis, senescence, and growth arrest are induced.

In addition, three of the five Hex2Cer isoforms were found to be upregulated. Kang-Yu Peng has reported a positive correlation between Hex2Cer and NASH, and increased Hex2Cer levels were detected in human liver cancer tissues. Also, animal studies showed that pharmacological inhibition of Hex2Cer synthase improved steatosis. These observations provide a strong rationale for further investigation of the role of Hex2Cer in the transition from NAFLD to NASH and possibly hepatocellular carcinoma.

### Other Lipid Changes

Compared to the control group, the AcOEtE group showed a significant increase in sterol esters, including cholesterol ester (ChE), Zymosterol ester (ZyE), and Stigmasteryl ester (StE). Excessive ChE can lead to fat accumulation in the liver, which can cause abnormal liver function in the long term. It was shown that C57BL/6J mice fed a high-fat, high-cholesterol diet developed severe hepatic steatosis, massive inflammation, and perisinusoidal fibrosis, which was associated with adipose tissue inflammation and reduced plasma lipocalin levels. In contrast, mice fed a high-fat diet without cholesterol developed only simple steatosis (El Kasmi et al., 2013).

## Conclusion

In this study, the histological assessment was combined with physicochemical index analysis to demonstrate that ethyl acetate extracts of *Rhizoma Paridis* (AcOEtE) induced liver injury and hepatic fibrosis in zebrafish larvae. Lipidomic analysis based on Q-Exactive HF-X mass spectrometer combined with pathway analysis strategies revealed that AcOEtE induced changes in the lipid profile of zebrafish larvae, mainly significant changes in glycerophospholipid metabolites containing long-chain polyunsaturated fatty acids (LCPUFA), accompanied by changes in the sphingolipid pathway. The significant changes in glycerophospholipids were not only in their subclasses such as PE, PC, and CL but also in the composition of lipids containing long-chain polyunsaturated fatty acids (ω-3 fatty acids and ω-6 fatty acids). By analyzing different metabolites, we found that the mechanism of AcOEtE induced hepatic fibrosis was closely related to the glycerophospholipid-mediated inflammatory response as well as the mitochondrial metabolism involved in CL. Based on the reproducibility of the quality control results, the method was considered reliable. In conclusion, this study revealed the mechanism of AcOEtE leading to liver injury and hepatic fibrosis from the lipid molecular level, which provides new ideas for further research on the safe clinical use of *Rhizoma Paridis*.

## Data Availability

The original contributions presented in the study are included in the article/Supplementary Material, further inquiries can be directed to the corresponding authors.

## Abbreviations

AA, arachidonic acid; AcOEtE, ethyl acetate extracts of *Rhizoma Paridis;* ALF, acute liver failure; ALT, alanine transaminase; AST, aspartate transaminase; Cer, Ceramide; CL, cardiolipin; DHA, docosahexaenoic acid; DCME, dichloromethane extracts of *Rhizoma Paridis;* DMePE Dimethyl-phosphatidyl ethanolamine; EPA, eicosapentaenoic acid; FC, fold change value; H&E, hematoxylin and eosin; HSC hepatic stellate cells; LC-PUFA, Long-chain polyunsaturated fatty acids; LPI, lysophosphatidylinositol; MTBE methyl-tert-butyl ether; NAFLD, Non-alcoholic fatty liver disease; NASH, Non-alcoholic steatohepatitis; PBS, phosphate-buffered solution; PC, glycerophosphocholine; PCA, principal component analysis; PE, glycerophosphoethanolamine; PEE, petroleum ether extracts of *Rhizoma Paridis;* PG, glycerophosphoglycerol; PLS-DA, partial least squares discriminate analysis; PI glycerophosphoinositol; PS, glycerophosphoserine; PUFA, polyunsaturated fatty acid; QC, quality control; qPCR, quantitative real-time polymerase chain reaction; Sph, sphingosine; TNF, tumor necrosis factor; WSBE, water-saturated n-butanol extracts of *Rhizoma Paridis;* WE, water extracts of *Rhizoma Paridis.*
