# Supplementary material for: Lipidomics Indicates the Hepatotoxicity Effects of EtOAc Extract of Rhizoma Paridis
Source: Front Pharmacol. 2022 Feb 8;13:799512. doi: 10.3389/fphar.2022.799512 (PMC8861452; doi:10.3389/fphar.2022.799512)
Supplement: Supplementary file 1 [file DataSheet1.docx]

Supplementary Material

**Components analysis of EtOAc extract of *Rhizoma Paridis*.**

We analyzed the composition of dried EtOAc extracts of ***Rhizoma Paridis* (AcOEtE)**. The specific method is as follows: UHPLC-Q-Exactive Orbitrap MS analysis was performed on the U3000 series Ultra-High-Performance Liquid Chromatography (UHPLC, California, USA), including a binary gradient pump, in-line degasser, autosampler, column temperature chamber, 4°C thermostat and diode array detector with Chromeleon workstation software. Chromatographic separation was performed at 35 °C using an ACQUITY UPLC HSS T3 column (2.1 × 100 mm, 1.8 μm). The mobile phase consisted of A (H2O, containing 0.1% formic acid) and B (CH_3_CN, containing 0.1% formic acid). The elution gradients were: 0~10min, 0~30%B, 10~25min, 30%~40%B, 25~30min, 40%~45%B, 30~40min, 50%~70%B, 40~45min. 70%~100%B, 45~60min, 100%B, 60~60.5min, 100%~0%%B, 60.5-70min, 0%B. The flow rate was 0.2mL/min and the column temperature was 35°C. The injection volume was 5μL.The mass spectrometer was operated in both positive and negative ion modes. The detailed Q-Exactive mass parameters were shown in Table S1.

We identified a total of 98 compounds in the ethyl acetate extracts of ***Rhizoma Paridis***, including Polyphyllin I, Polyphyllin VI, Polyphyllin E, Dioscin, Diosgenin, Protodioscin, Gracillin, etc. (Table S2). The EtOAc extracts were qualitatively analyzed using the standard comparison method based on the characteristics of the same compounds with similar retention times and product ions under the same chromatographic conditions. The components in the extracts were determined by comparing the retention time and product ion information of the sample peaks and the standard peaks.

**Supplementary Table**

**Table S1.** The parameters of Q- Exactive mass

| **Q-Exactive mass parameters** | **Details** |
| --- | --- |
| Scan type | Full MS-ddMS^2^ |
| Scan range | 100 to 1500 |
| Spray Voltage(kV) | 3.5/-3.2 |
| Chrom.peak width (FWH) | 15 |
| Resolution | 70000 |
| AGC target | 3.00E+06 |
| Maximum IT (ms) | 100 |
| Sheat gas | 40/45 |
| Aux gas flow | 11/10 |
| Sweep gas | 0 |
| Capillary temperature (℃) | 350 |
| S-lens RF level | 55 |
| Aux gas heater temperature (℃) | 220 |

**Table S2.** The parameters of UHPLC-Q Exactive HF-X mass

| **Q-Exactive mass parameters** | **Details** |
| --- | --- |
| Scan type (m/z) | 200-2000 |
| Sheath gas flow rate (psi) | 60 |
| Sheath gas flow rate (psi) | 20 |
| Aux gas flow rate (psi) | 20 |
| Aux gas heater temp (℃) | 370 |
| IonSpray Voltage Floating (ESI+) (V) | +3000 |
| IonSpray Voltage Floating (ESI-) (V) | -3000 |
| Normalized collision energy (V) | 20-40-60 |

**Table S3. Primer sequences used in mRNA quantitation by reverse transciption-polymerase chain reaction.**

| Gene | Forward primers | Reverse primers |
| --- | --- | --- |
| Fabp11a | AGGTTGGAAACCGGACCAA | CCACCACATCACCCATCTTGC |
| Fabp7a | TGTGGAGACTGAACTCAGCG | CCCACCACCATCATTGACATTC |
| FASN | AGTGATGTCAGCACCCTGGA | GGTGAACCAGGAACAAAGGC |
| PPARG | TCAGAAAGCTTCACTCTCCGC | GGCACTCGATGTTCAGTGAGG |
| FADS2 | TTCAGAGATCAGCGATGGGT | TGGTTGTAGGGCAGATGCTTG |
| CPT1 | CACAGGTCTGTGGATTGCCA | GGCCAGGATCCTCTCCATCT |
| CD36 | GCGATGTACAGGCAGTTCTGG | CACCTTGCCAGCGTTCAAT |
| LCLAT | GATTTGCTGCGGGACTCAGA | ATGTTGCTCATGTGTGTGCG |
| Taz | GAACCTGGTTGATGAGCGTCC | CTCTGCCTGGGCTTTCAAACT |
| LPCAT4 | GTGTGTGTCACTCGAGCACT | CATGAGCATCTGAGGCCAGT |
| TNFα | GTGGCACCTGTACCGGTTAAT | CGTAGTTGTTCTCGATGTCGC |
| IL6ST | GCTCCAATGTTACAGTTCG | GCTTTCACAGGAGGCTTA |
| PI3K | ACATGGCTCTGCAAGATGCT | GGAGGCATCTCGGACCAAAA |
| AKT | TCGGCAGGTGTCTTCTCAAT | ACCCATTGCCATACCACGAG |
| mTOR | GGGTCAGCTTCACCAACAGT | TTGCGATCATGCTCCGTTGA |

| **Table S4.** The 99 chemical compounds of the ethyl acetate extract of *Rhizoma Paridis* | | | | | |
| --- | --- | --- | --- | --- | --- |
| RT [min] | Formula | Name | Calc. MW | mzVault Best Match | Group Area |
| 21.525 | C27 H44 O7 | Hydroxyecdysone | 480.30832 | 82.6 | 22499381619 |
| 30.686 | C18 H34 O5 | (15Z)-9,12,13-Trihydroxy-15-octadecenoic acid | 330.24031 | 82.1 | 9691879451 |
| 43.242 | C16 H22 O4 | Dibutyl phthalate | 278.1518 | 95.3 | 7705407482 |
| 1.718 | C12 H22 O11 | α, α-Trehalose | 342.11584 | 95 | 7510274660 |
| 31.721 | C51 H82 O21 | Pseudoprotodioscin | 1030.53389 | 87.1 | 5153515026 |
| 36.864 | C44 H70 O16 | Polyphyllin I | 854.46573 | 84.9 | 3479660099 |
| 36.868 | C27 H42 O3 | Diosgenin | 396.30284 | 83.3 | 2239505191 |
| 36.867 | C27 H42 O3 | Diosgenin | 414.31328 | 89 | 2082286831 |
| 50.253 | C24 H38 O4 | Bis(2-ethylhexyl) phthalate | 390.27676 | 93.2 | 2035558950 |
| 35.838 | C51 H82 O20 | Polyphyllin E | 1014.53928 | 86.8 | 1779771364 |
| 32.006 | C45 H72 O17 | Pennogenin 3-O-β-chacotrioside | 884.47661 | 86.6 | 1378180088 |
| 21.527 | C21 H30 O4 | Corticosterone | 346.21425 | 78.3 | 1376481228 |
| 36.41 | C45 H72 O16 | Dioscin | 868.48174 | 84.5 | 1123351628 |
| 12.118 | C10 H13 N5 O4 | Adenosine | 267.09682 | 94.8 | 900994478.2 |
| 3.382 | C6 H6 N2 O | Nicotinamide | 122.04805 | 98.5 | 869638080.5 |
| 1.354 | C6 H13 N O4 | 1-Deoxynojirimycin | 163.08453 | 81.4 | 585908426.2 |
| 24.698 | C33 H52 O8 | Diosgenin glucoside | 576.36639 | 83.8 | 483977898.9 |
| 24.571 | C45 H72 O17 | Gracillin | 884.47655 | 78.2 | 401379408.1 |
| 24.538 | C51 H84 O22 | Protodioscin | 1048.54475 | 84.7 | 360973414.6 |
| 36.864 | C19 H28 O2 | Dehydroepiandrosterone (DHEA) | 270.19821 | 81.5 | 359927258.1 |
| 5.925 | C9 H12 N2 O6 | Uridine | 244.06937 | 94.5 | 348407096.4 |
| 18.928 | C22 H30 O14 | Sibiricose A5 | 518.16332 | 90 | 265466875.6 |
| 32.753 | C39 H62 O13 | Polyphyllin VI | 738.41896 | 92 | 249196866.1 |
| 50.403 | C22 H42 O4 | Bis(2-ethylhexyl)adipate | 370.30823 | 91.3 | 244367964.3 |
| 29.077 | C18 H28 O3 | 9S,13R-12-Oxophytodienoic acid | 292.20378 | 76.4 | 219880640.6 |
| 4.724 | C8 H11 N O3 | Pyridoxine | 169.07395 | 91.8 | 171102763 |
| 31.723 | C27 H42 O4 | Hecogenin | 430.30819 | 72.1 | 170206039.8 |
| 1.489 | C5 H13 N O | Choline | 103.09966 | 94.4 | 156481365.3 |
| 29.075 | C18 H26 O2 | 19-Nortestosterone | 274.19332 | 79.8 | 151059142.5 |
| 2.946 | C6 H5 N O2 | Nicotinic acid | 123.03216 | 86.7 | 137827390.9 |
| 17.858 | C11 H15 N5 O3 S | 5'-S-Methyl-5'-thioadenosine | 297.0896 | 94.9 | 127233792.2 |
| 18.938 | C16 H18 O8 | 4-Methylumbelliferyl-α-D-glucopyranoside | 338.10039 | 79.4 | 123904090.1 |
| 43.261 | C18 H32 O7 | Citroflex 4 | 360.21468 | 87.9 | 105611591.5 |
| 2.802 | C5 H5 N5 | Adenine | 135.05445 | 95.6 | 95817279.79 |
| 40.461 | C18 H30 O2 | α-Eleostearic acid | 278.22478 | 90.7 | 89222435.32 |
| 4.553 | C4 H6 O4 | Succinic acid | 118.0265 | 91.6 | 81733226.03 |
| 23.586 | C34 H42 O19 | 3',6-Disinapoyl sucrose | 754.23182 | 88.7 | 80434293.48 |
| 23.464 | C24 H32 O6 | Arenobufagin | 416.21993 | 76.1 | 72899724.87 |
| 31.25 | C19 H24 O2 | 1,4-Androstadiene-3,17-dione | 284.17775 | 86.6 | 72580376.01 |
| 23.215 | C9 H16 O4 | Azelaic acid | 188.10474 | 82.8 | 72573951.97 |
| 3.77 | C5 H7 N O3 | D-(+)-Pyroglutamic Acid | 129.04262 | 73.1 | 71717286.58 |
| 21.983 | C10 H10 O4 | Ferulic acid | 194.05801 | 85.2 | 71223934.99 |
| 2.106 | C4 H6 O5 | L-(-)-Malic acid | 134.02138 | 98.2 | 67547688.83 |
| 21.343 | C9 H8 O3 | 3-Coumaric acid | 164.0473 | 90.8 | 66973778.52 |
| 18.084 | C14 H20 O7 | Salidroside | 300.12067 | 87.7 | 62716122.94 |
| 36.904 | C18 H39 N O2 | 2-Amino-1,3-octadecanediol | 301.2982 | 70.1 | 58116289.86 |
| 18.905 | C8 H8 O4 | 3-Methoxysalicylic acid | 168.0421 | 96.7 | 57272716.38 |
| 16.519 | C16 H16 F3 N O | Norfluoxetine | 295.11705 | 78.2 | 53816679.3 |
| 16.943 | C9 H17 N O5 | Pantothenic acid | 219.1106 | 77.8 | 50923281.51 |
| 22.572 | C28 H32 O16 | Narcissoside | 624.16943 | 86.7 | 50804134.26 |
| 50.273 | C16 H22 O4 | Dibutyl phthalate | 278.15181 | 91.6 | 45973367.94 |
| 18.941 | C10 H8 O3 | 7-Methoxycoumarin | 176.04747 | 70.8 | 43219191.62 |
| 1.901 | C12 H22 O11 | D-(+)-Maltose | 364.09853 | 78.3 | 42800027.79 |
| 20.096 | C7 H6 O2 | Benzoic acid | 122.03669 | 79.8 | 41119756.72 |
| 35.602 | C24 H30 O6 | Bis(4-ethylbenzylidene)sorbitol | 414.20423 | 90.2 | 39164934.2 |
| 28.632 | C18 H30 O3 | 9-Oxo-ODE | 294.21944 | 77.6 | 35631511.02 |
| 24.799 | C51 H84 O23 | Protogracillin | 1064.53976 | 82.8 | 35428006.06 |
| 21.942 | C21 H24 O9 | Rhaponticin | 420.14196 | 82.7 | 34791066.77 |
| 46.029 | C18 H30 O2 | α-Linolenic acid α | 278.22478 | 88.4 | 34258054.81 |
| 49.295 | C18 H37 N O | Stearamide | 283.28758 | 80.7 | 32668848.74 |
| 21.339 | C8 H14 O4 | Suberic acid | 174.08898 | 93.6 | 32402734.94 |
| 31.803 | C17 H34 O2 | Methyl palmitate | 287.28236 | 76.1 | 32220221.09 |
| 33.108 | C17 H26 O4 | 6-Gingerol | 294.18305 | 82.8 | 31432891.37 |
| 37.82 | C21 H38 O4 | 1-Linoleoyl glycerol | 354.27711 | 75.4 | 31195455.39 |
| 19.093 | C23 H32 O15 | Sibiricose A6 | 548.17421 | 91 | 31112465.01 |
| 3.493 | C6 H7 N O | Nicotinyl alcohol | 109.05286 | 70.4 | 31059591.31 |
| 1.51 | C6 H6 O3 | Maltol | 126.03176 | 81.5 | 29980547.87 |
| 19.38 | C7 H12 O4 | 3-Methyladipic acid | 160.07336 | 93.9 | 29130678.11 |
| 36.873 | C27 H40 O3 | Testosterone cypionate | 412.29753 | 84.2 | 26888704.23 |
| 23.293 | C21 H24 O10 | Phloridzin | 436.1367 | 87.8 | 26843375.13 |
| 20.889 | C16 H22 O9 | 3-[2-(β-D-Glucopyranosyloxy)-4-methoxyphenyl]propanoic acid | 358.12626 | 70.9 | 26319456.96 |
| 30.83 | C18 H15 O P | Triphenylphosphine oxide | 278.0862 | 77.5 | 25786237.48 |
| 21.596 | C28 H32 O16 | Isorhamnetin-3-O-nehesperidine | 624.16841 | 79.5 | 25532681.92 |
| 21.934 | C15 H14 O4 | Rhapontigenin | 258.08934 | 86.3 | 24626180.22 |
| 1.36 | C6 H14 N4 O2 | DL-Arginine | 174.11154 | 87.9 | 23664186.56 |
| 21.261 | C8 H8 O3 | 2-Hydroxy-4-methoxybenzaldehyde 4 | 152.04749 | 77.8 | 22946481.44 |
| 28.423 | C12 H18 O2 | Sedanolide | 194.13075 | 75.7 | 21319364.58 |
| 20.937 | C8 H8 O | Acetophenone | 120.05756 | 87.2 | 21282461.26 |
| 1.503 | C5 H12 O5 | L-(-)-Arabitol | 152.06833 | 80.1 | 21168739.23 |
| 22.666 | C16 H12 O7 | Isorhamnetin | 316.05849 | 89.7 | 21119580.14 |
| 23.406 | C27 H40 O4 | Hydroxyprogesterone caproate | 428.2928 | 71.3 | 20514470.45 |
| 3.393 | C5 H10 O5 | D-(-)-Lyxose | 150.05269 | 77.8 | 20472018.26 |
| 30.686 | C9 H14 | 1,2,3,4-Tetramethyl-1,3-cyclopentadiene | 122.10951 | 89.4 | 20428273.73 |
| 50.243 | C8 H6 O4 | Phthalic acid | 166.02641 | 87.7 | 17300735.89 |
| 23.246 | C30 H32 O14 | Methyl (1S,4aS,7S,7aS)-1-(β-D-glucopyranosyloxy)-4'-[(1S)-1-{[(2E)-3-(4-hydroxyphenyl)-2-propenoyl]oxy}ethyl]-5'-oxo-4a,7a-dihydro-1H,5'H-spiro[cyclopenta[c]pyran-7,2'-furan]-4-carboxylate | 616.17961 | 77.7 | 13268857.84 |
| 24.443 | C26 H42 O10 | 16-(Hexopyranosyloxy)-7-hydroxy-8,9-epoxypimaran-18-oic acid | 514.27754 | 80.2 | 13042942.85 |
| 22.508 | C21 H20 O11 | Astragalin | 448.10045 | 82 | 13012977.14 |
| 23.035 | C16 H14 O6 | Hesperetin | 302.07903 | 85.9 | 12624118.21 |
| 18.535 | C7 H12 O5 | 2-Isopropylmalic acid | 176.06834 | 89.5 | 12599402.11 |
| 2.809 | C4 H4 N2 O2 | Uracil | 112.02724 | 90.2 | 12539484.79 |
| 17.993 | C7 H13 N O3 | N-Acetylvaline | 159.08938 | 90.8 | 11332529.5 |
| 23.293 | C15 H14 O5 | Phloretin | 274.08397 | 86.8 | 11256525.37 |
| 29.452 | C12 H22 O4 | Dodecanedioic acid | 230.15156 | 70.6 | 10899517.67 |
| 20.291 | C8 H15 N O3 | N-Acetyl-L-leucine | 173.10515 | 87.3 | 10233568.29 |
| 19.089 | C11 H12 O5 | Sinapinic acid | 206.05803 | 80.7 | 8980694.787 |
| 25.163 | C10 H18 O4 | 3-tert-Butyladipic acid | 202.12045 | 71.3 | 8714818.697 |
| 24.26 | C16 H14 O5 | Sakuranetin | 286.08406 | 78.3 | 8205396.19 |
| 21.227 | C15 H20 O8 | 4-Acetyl-3-hydroxy-5-methylphenyl β-D-glucopyranoside | 374.12126 | 71.8 | 7841208.914 |

**Figure S1.** The total ion chromatograms (TIC) of the QC samples in negative and positive modes.

**
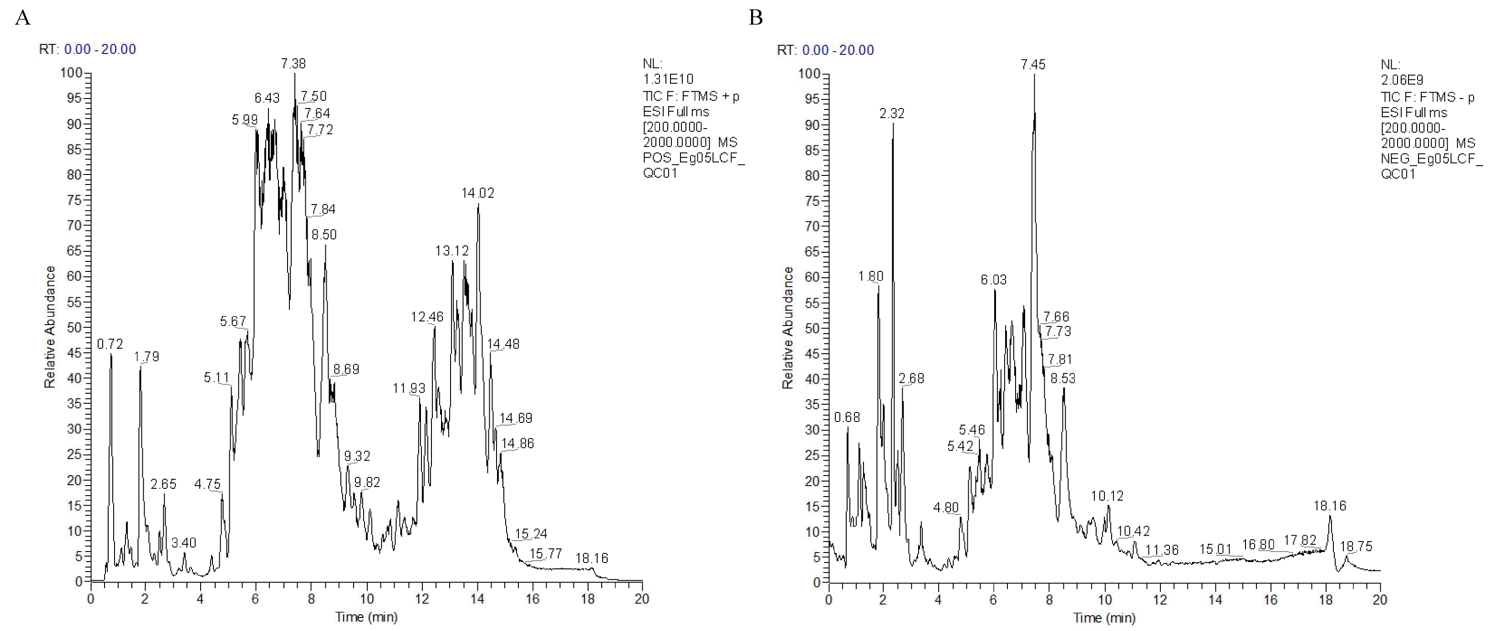
**

**Figure S1** The Total Ion Chromatogram of QC samples. **(A)** The Total Ion Chromatogram of QC samples (ESI +). (ESI+) represents the positive ion detection mode, in which the mass analyzer scans only positive charged ions and filters out negative charged ions to obtain positive charged ions information during the detection process; **(B)** The Total Ion Chromatogram of QC samples (ESI-). (ESI-) denotes the negative ion detection mode, in which the mass analyzer scans only negative charged ions and filters out positive charged ions, thus obtaining the information of negative charged ions.

**Figure S2.** The heatmap of 325 lipids in the control and AcOEtE groups

**
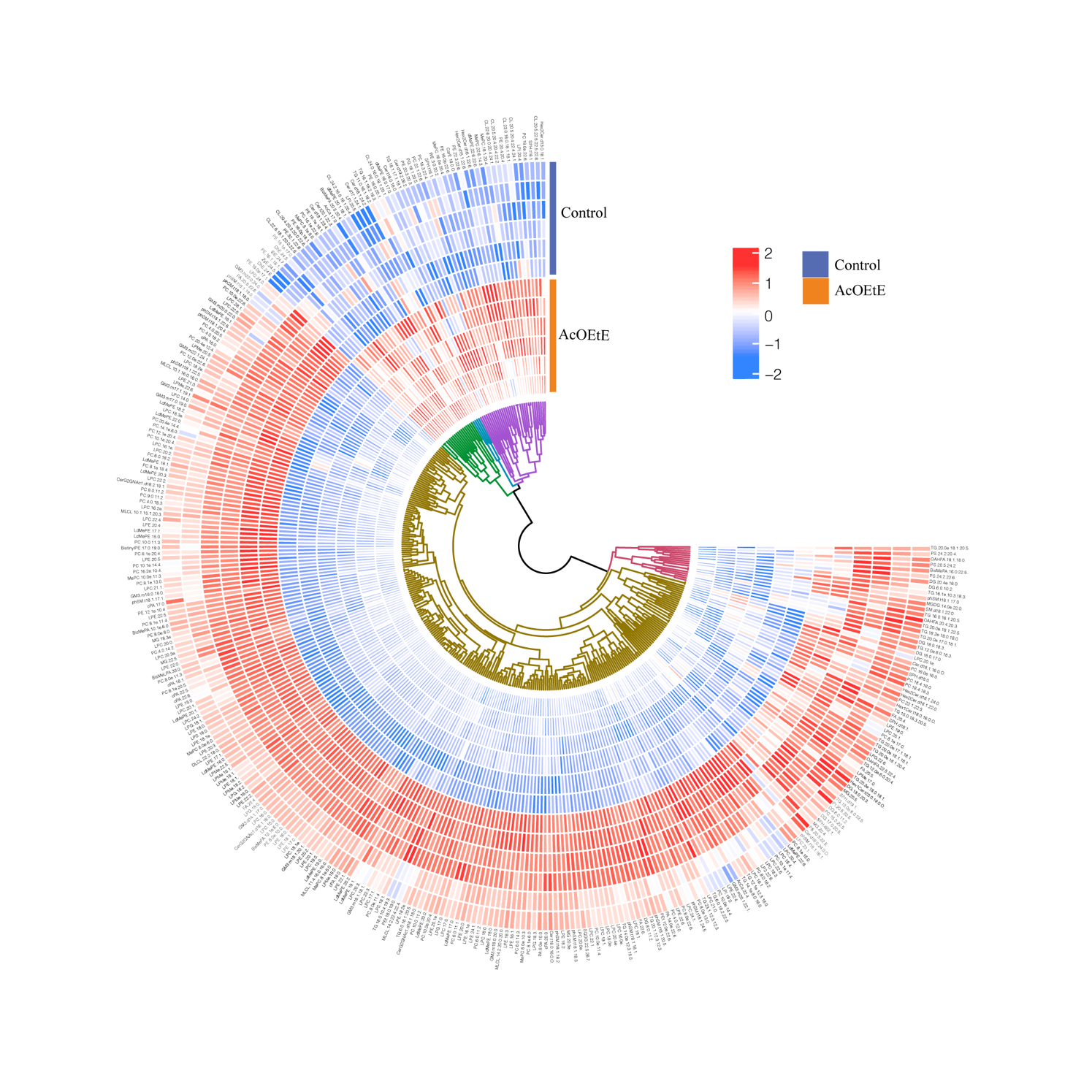
**

**Figure S2** The heatmap of 325 lipids in the control and AcOEtE groups. The red and blue colors indicate the increased and decreased levels, respectively.
